# Retinal glial cells in glaucoma and age-related retinal diseases: Inflammatory responses, disease transitions, and translational perspectives

**DOI:** 10.4103/NRR.NRR-D-25-01905

**Published:** 2026-02-05

**Authors:** Akanksha Salkar, Viswanthram Palanivel, Devaraj Basavarajappa, Benjamin Heng, Angela Schulz, Vivek Gupta, Stuart Graham, Mehdi Mirzaei, Yuyi You

**Affiliations:** 1 Macquarie Medical School, Faculty of Human Health and Medical Science, Macquarie University, Sydney, NSW, Australia; 2 Save Sight Institute, University of Sydney, Sydney, NSW, Australia

**Keywords:** biomarker, blood–retinal barrier, cytokine, glaucoma, gliosis, humanized model, neurodegeneration, neuroinflammation, oxidative stress, proteomics, retina

## Abstract

Microglia, Müller cells, and astrocytes play a crucial role in maintaining retinal structure, homeostasis, and neuronal function. In disease, they undergo reprogramming that drives chronic inflammation and neurodegeneration. Unique to the retina, these glial cells occupy specialized niches and interact closely with the blood–retinal barrier, creating distinct vulnerabilities. We summarized the glial activation mechanisms, shared triggers, including oxidative stress, metabolic dysfunction, aging, and systemic inflammation, as well as key pathways, such as nuclear factor kappa-B, mitogen-activated protein kinase, Janus kinase/signal transducer and activator of transcription, the inflammasome, and the complement system. Disease-specific responses in glaucoma, age-related macular degeneration, diabetic retinopathy, and vascular occlusions were compared, highlighting the heterogeneity of gliosis and its impact on neuronal and vascular pathology. We also discussed emerging human-derived platforms alongside proteomics approaches, highlighting their utility for mechanistic insights and discovering biomarkers. Despite advances, critical gaps remain in understanding glial–glial interactions and in developing robust models focused on glia. Despite these advances, major gaps remain in our understanding of glial–glial communication, state transitions, and their temporal relationship to neurodegeneration. Moreover, the lack of experimental models explicitly designed to interrogate glial biology continues to limit translational progress. Addressing these challenges will be essential to reposition glial cells as central drivers of retinal disease rather than secondary responders. A strategic shift toward glia-centered models, integrative multi-omics analyses, and human-relevant systems holds promise for advancing biomarker discovery and developing targeted therapeutic strategies that aim to modulate glial dysfunction and preserve vision.

**Facts**

• Retinal glial cells are active drivers of disease, not passive responders in disease pathogenesis, yet mechanisms remain unexplored.

• Glial phenotypes undergo disease- and stage-specific transitions, with distinct inflammatory and neuroprotective states emerging at different disease stages.

• Evidence across retinal diseases suggests that glial-driven inflammatory changes may occur before measurable neurodegeneration, positioning glia as early indicators of disease activity.

• Multi-omic investigations at the cellular level using human-derived tissues and disease models offer significant mechanistic insight; however, such studies remain limited.

• Modulating glial activation states offers opportunities for biomarker discovery and therapeutic intervention that may complement or surpass neuron-centric approaches.

**Open questions**

• Can distinct microglial or astrocytic phenotypes be selectively targeted to promote neuroprotection without suppressing essential immune functions?

• Can disease-associated glial states revert to homeostatic phenotypes, and at what stage of the disease does this occur?

• Are certain glial responses conserved across retinal diseases, while others remain disease-specific, and could they help in precision medicine strategies?

• Can circulating or ocular glial markers be developed for early diagnosis, prognosis, and treatment monitoring using fully validated cohorts in humans?

## Introduction

Retinal glial cells, comprising Müller glia (MG), astrocytes, and microglia, play central roles in maintaining neuronal homeostasis, providing metabolic support, and ensuring the structural integrity of the retina (Fletcher et al., 2008; Reichenbach, 2016). Under normal conditions, MG regulate ion and water balance, recycle neurotransmitters, and support synaptic function (Bringmann et al., 2006); astrocytes maintain the blood–retinal barrier (BRB), modulate energy supply, and structurally support retinal ganglion cell (RGC) axons (Cullen and Sun, 2023); microglia serve as immune sentinels, constantly surveying the retinal landscape (Rathnasamy et al., 2019). Beyond their physiological functions, these cells act as the primary mediators of neuroinflammation within the retinal microenvironment. Once regarded merely as supportive to neurons, glial cells are now increasingly implicated in the pathological course of retinal degenerative diseases, including glaucoma, age-related macular degeneration (AMD), diabetic retinopathy (DR), and retinal vascular occlusion.

This activation of glial cells leads to the release of effector molecules, such as cytokines and chemokines, and eventually leads to the recruitment of peripheral cells through the dysregulated BRB, which can be defined as neuroinflammation in the brain. Although neuroprotective initially, sustained inflammation may be detrimental due to a lack of neuronal tissue regeneration (Adornetto et al., 2019). Glial activation occurs on detection of cellular distress signals triggered by diverse stressors, including aging, oxidative stress, mitochondrial dysfunction, metabolic imbalance, and mechanical strain (Wei et al., 2019). Such stimuli disrupt homeostatic signaling, leading to reactive gliosis, a state characterized by morphological remodeling, altered gene expression, and the release of cytokines, chemokines, and other inflammatory mediators (Reichenbach, 2016). While these early responses can be protective, aiding debris clearance and tissue repair, prolonged or dysregulated activation can become maladaptive, exacerbating neuronal injury and promoting chronic neuroinflammation.

In glaucoma, for example, stressors such as elevated intraocular pressure (IOP) and age-related vascular and metabolic changes initiate early glial reactivity in the retina and optic nerve head (ONH) (Tezel, 2021, 2022). Emerging evidence suggests that, in addition to the individual susceptibility of RGCs to glaucomatous injury, the responses of surrounding glia are also crucial for the fate of these neurons (Zhao et al., 2021; Amato et al., 2023; Batsuuri et al., 2025). Glia-driven neuroinflammation is detectable at multiple injury sites, ranging from the retina to upper brain centers, and at various time points, from the early to late stages of RGC degeneration. However, owing to the immune-privileged status of the retina and optic nerve, glia-driven neuroinflammation, as opposed to classical inflammation, is somehow dampened to an intermediate state called parainflammation (Medzhitov, 2008). Although initially aimed at neuroprotection, persistent glial activation in glaucoma contributes to excitotoxicity, oxidative damage, and RGC degeneration. Similar mechanisms are increasingly recognized in other chronic retinal disorders, including AMD and DR. This highlights the importance of understanding glial biology as both a driver and a potential therapeutic target in retinal neurodegeneration. This review examines the roles of glial cells in maintaining retinal homeostasis and their activation during disease, focusing particularly on glaucoma. We also made comparisons with other retinal diseases, including AMD, DR, and retinal vascular occlusions (RVO). We discussed the use of human models to study ocular diseases and glial interactions, as well as high-throughput proteomic approaches for identifying glaucoma progression biomarkers. While emphasizing the usefulness of humanized models and proteomics, the review also highlighted the need for multi-parametric studies that explicitly incorporate glial cells into the analysis.

## Search Strategy

A literature search was conducted by searching PubMed/MEDLINE, Web of Science, and Scopus for peer-reviewed articles published up to 2025, with a focus on studies from 2016 onwards to reflect advances in technology. Only English-language articles were included, and additional relevant studies were identified through manual screening of reference lists from key reviews and seminal primary studies. Search terms were organized into thematic domains, encompassing retinal glial cell types (microglia, astrocytes, and Müller glia), activation states and phenotypes, neuroinflammatory and neuroprotective processes, biomarker discovery, and blood–retinal barrier (BRB) integrity, combined with disease-specific terms such as glaucoma, age-related macular degeneration, diabetic retinopathy, and retinal vascular occlusions.

For proteomics studies, the proteins significantly enriched in each study were extracted, organized by tissue, and analyzed using Metascape (https://metascape.org/gp/#/main/step1) to identify shared enriched pathways (**Additional Table 1**).

**Additional Table 1:** Summary of glial cell responses in health and disease

Glial cell	Homeostatic function	Disease	Reactive/inflammatory changes	Morphological changes	Impact on pathogenesis	Key pathway/mediator	Reference
Microglia	Immune surveillance, debris clearance, neurotrophic support	Glaucoma	Hypertrophic/amoeboid, upregulation of Ibal, CD68, HLA-DR; pro-inflammatory cytokines	Ramified → hypertrophic/amoeboid → rod-shaped or satellite near axons	Amplify neuroinflammation, promote axonal degeneration and synaptic loss	TNF-α, IL-1β, IL-6, complement (C1 q, C3, and C5), NLRP3 inflamma some, cGAS-STING, MAPK, NF-κB, and JAK/STAT	Colonna and Butovsky, 2017 Savage et al., 2019; Yu et al., 2021 Salkar et al., 2024, 2025
		DR	Hypertrophy, perivascular clustering, pro-inflammatory/pro-angiogenic mediator release	Hypertrophic, clustering around vessels, infiltration of retinal layers	Drive vascular infiammation, leukostasis, and neurovascular unit dysfunction	TNF-α, IL-1β, IL-6, VEGF, MMPs, NF-kB, and P2 receptor signaling	Yoshida et al., 1999 Grigsby et al., 2014; Yu et al., 2015
		AMD	Recruitment to the subretinal space, pro-inflammatory activation	Hypertrophic/amoeboid, cluster around drusen or at GA lesions	Drive local chronic infiammation, complement-mediated damage, photoreceptor/RPE degeneration	IL-1β, IL-6, TNF-α, complement (C3a, and C5a), NLRP3 inflamma some	Nita et al., 2014; Fletcher, 2020 Ascunce et al., 2023
		Retinal vascular occlusion	Rapid activation with cytokine/ROS release (RAO); sustained perivascular clustering and macrophage infiltration (RVO)	Not well characterized	Exacerbate acute ischemic neuronal death (RAO) and promote chronic vascular leakage, edema, and neovascularization (RVO)	TNF-α, IL-1β, IL-6, ROS, NF-κB, TLR signaling, HIF-1α-VEGF	Jovanovic et al., 2020 Zhang et al., 2025 b
Astro cytes	Structural/metabolic support to RGC axons, gap-junction communication, anti-angiogenic/metabolic support	Glaucoma	GFAP upregulation, redistribution, reactive A1/A2 phenotypes	Stellate → hypertrophic, peripapillary redistribution, elongated bundles in NFL	Alter ECM promotes neuroinflammation, can be neurotoxic or neuroprotective	TNF-α, NO, endothelin signaling, ECM remodeling	Liddelow and Barres, 2017 Lukowski et al., 2019 McGrady et al., 2021; Cooper and Calkins, 2024
		DR	Reduced density, retracted processes, COX-2/PGE2 induction	Retraction from axonal bundles, reduced coverage in peripapillary/peripheral retina	Contribute to vascular and neuronal dysfunction	EGFR/TGF-α, COX-2, VEGF	Zhang and Neufeld, 2005; Yu et al., 2015
		AMD Retinal vascular occlusion	Less characterized; may contribute to inflammation N/A	Not well characterized Not well characterized	Limited data on the contribution to pathogenesis N/A	N/A N/A	
Müller cells	Metabolic support, glutamate clearance, lactate shuttling, homeostatic support	Glaucoma	Gliosis, TRPV4/Piezo1 activation, impaired glutamate/K^+^ buffering, oxidative stress	Process thickening, endfeet swelling, regional proliferation (peripheral > central)	Impair metabolic support and excitotoxicity worsen RGC injury	EAAT1 dysfuncti on, Kir4.1 reduction, oxidative stress pathways, Wnt/β-catenin	Daruich et al., 2018 Shinozaki and Koizumi, 2021 Pereiro et al., 2024
		DR	Hyperplasia, GFAP upregulation, VEGF, IL-1β, TNF-α secretion	Endfeet swelling, chromatin dispersion, lysosomal granules, peripheral proliferation	Promote vascular leakage, angiogenesis, and neuroinflammation	Wnt/β-catenin, p38 MAPK/NF-κB, RAGE-MAPK	Zong et al., 2010 Zhou et al., 2014 Yang et al., 2022
		AMD Retinal vascular occlusion	Less characterized Acute gliosis, oxidative stress, impaired glutamate/K^+^ buffering (RAO); sustained gliosis with VEGF/cytokine release, BRB breakdown (RVO)	Not well characterized Endfeet swelling, thickened processes, proliferative changes	Limited data on the contribution to pathogenesis Accelerate excitotoxic death and infarction (RAO); drive vascular leakage, macular edema, and neovascularization (RVO)	N/A VEGF, HIF-1α, PDGF, Angiopoietins, NF-κB, oxidative stress pathways, Wnt/β-catenin	Zhou et al., 2014 Zhou et al., 2023 Zhang et al., 2025 b

AMD: Age-related macular degeneration; BRB: blood-retinal barrier; cGAS-STING: cyclic GMP-AMP synthase-stimulator of interferon genes; COX-2: cyclooxygenase-2; DR: diabetic retinopathy; EAAT1: excitatory amino acid transporter 1; ECM: extracellular matrix; GA: geographic atrophy; GFAP: glial fibrillary acidic protein; HIF-1α: hypoxia-inducible factor 1 alpha; IL-1β: interleukin-1 beta; IL-6: interleukin-6; JAK/STAT : Janus kinase/signal transducer and activator of transcription; Kir4.1 : inward-rectifying potassium channel 4.1; MAPK: mitogen-activated protein kinase; MMP: matrix metalloproteinase; NFL: nerve fiber layer; NF-κB: nuclear factor kappa B; NLRP3: NOD-like receptor pyrin domain-containing 3 inflammasome; NO: nitric oxide; PDGF: platelet-derived growth factor; Piezol: mechanosensitive ion channel Piezol; RAGE: receptor for advanced glycation end-products; RAO: retinal artery occlusion; RGC: retinal ganglion cell; RVO: retinal vein occlusion; TNF-α: tumor necrosis factor alpha; TRPV4: transient receptor potential vanilloid 4; VEGF: vascular endothelial growth factor; Wnt/β-catenin: wingless/β-catenin signaling.

## Types and Functions of Retinal Glial Cells

### Müller glia

MG are retina-specific glial cells that span the entire thickness of the retina. The cell body of an MG localizes in the inner nuclear layer and bidirectionally extends its processes toward the inner and outer layers of the retina. In the inner and outer plexiform layers of the retina, the processes of MG enwrap synapses and form contacts with blood vessels (Shinozaki and Koizumi, 2021). They connect retinal neurons with surrounding compartments, including blood vessels, the vitreous body, and the subretinal space. Their roles include supplying trophic factors, clearing metabolic waste, maintaining ion and water homeostasis, preserving the inner BRB, regulating blood flow, and supporting synaptic activity through neurotransmitter recycling and precursor supply (Reichenbach and Bringmann, 2013). Currently, the many roles of MG in the regulation of retinal function in a diseased state remain unresolved and are the subject of intensive research (Bringmann and Wiedemann, 2012; Kobat and Turgut, 2020).

### Astrocytes

Astrocytes, the most prominent glial cells of the central nervous system, are central to the pathophysiology of glaucoma, as they represent the predominant cellular component of the ONH (Cullen and Sun, 2023). These fibrous glial cells provide structural and metabolic support across the visual pathway, spanning the retinal nerve fiber layer, ONH, and the myelinated optic nerve.

In the retina, astrocytes are confined to the ganglion cell and nerve fiber layers, where they form a dense mesh-like network in close association with blood vessels and RGC axons (Shinozaki and Koizumi, 2021). In primates, they exhibit two morphological forms: stellate astrocytes that interact with vasculature in the ganglion cell layer, and elongated astrocytes with processes aligned to axons in the nerve fiber layer. In the lamina cribrosa and prelaminar region of the ONH, astrocytes provide cellular support functions to the axons, form the interface between connective tissue surfaces, and surround blood vessels. In the furthest anterior region, a distinct population of fibrous retinal astrocytes tiles the vitreoretinal surface and is required for retinal vascularization during development. ONH astrocytes play a significant role in the structure of the lamina as they line the collagenous beams that form the mesh-like structure and insulate axons from connective tissue and vasculature (Cullen and Sun, 2023). These astrocytes are also believed to sense and respond directly to mechanical pressure. Functionally, astrocytes regulate ion balance, water and metabolite flux, neurotransmitter transmission, and synaptic plasticity. They also act as bioenergetic mediators between neurons and blood vessels, uniquely capable of synthesizing, storing, and mobilizing glycogen for neuronal support during high metabolic demand or stress (Liu et al., 2022; Cooper and Calkins, 2024).

### Microglia

Microglia form the first line of defense in the retina and brain, acting as the primary resident immune cells of the central nervous system while also contributing to retinal homeostasis (Wang et al., 2016; Wei et al., 2019). Derived from hematopoietic precursors, they enter the retina via the optic disc, ciliary body, iris, and retinal vessels (Chen et al., 2002). On entry to the retina, these precursors migrate parallel to the axon fascicles of the nerve ﬁber layer. Subsequently, it spreads through the retinal parenchyma. At this stage, these cells are activated, appearing as ameboid-shaped with short and broad branches, and play active roles during development. Finally, maturing into the so-called resting ramiﬁed microglia (Chen et al., 2002; Reichenbach, 2016).

In the adult retina, microglia exist as ramified cells with dendritic-like morphology that continuously survey their environment, thereby maintaining homeostasis, or as activated ameboid microglia in response to stress or injury. Although deemed resting, these cells are still involved in interactions with other neighboring cells. They are involved in neural plasticity, neurogenesis, synaptic pruning, maintenance of synaptic structure, and function (Rathnasamy et al., 2019). Microglia are also involved in clearing cellular debris by phagocytosis, which is essential for post-natal development and in the adult retina (Schafer et al., 2012; Rashid et al., 2019; Rathnasamy et al., 2019). As immune watchdogs, they mediate immune defense, tissue repair, and immunoregulation through complement activation, antigen presentation, and cytokine release. Inflammatory responses by retinal microglia include secretion of pro- or anti-inflammatory cytokines (tumor necrosis factor alpha [TNF-α], interleukin [IL]-1β, IL-3, IL-6, IL-10, IL-12, and IL-18), and express chemokine receptors (C–C chemokine receptor 1, C–C chemokine receptor 2, C–C chemokine receptor 5, C–X3–C chemokine receptor 1, C-X-C motif chemokine receptor 2, and C-X-C motif chemokine receptor 3) (Taylor et al., 2011; Kohno et al., 2014; Rutar et al., 2015). Another immune component, the inflammasome, is required for the maturation of cytokine precursors and, in turn, initiates apoptosis to induce inflammation. Retinal microglia have been found to express NOD-like receptor family pyrin domain-containing 3 (NLRP3), the most well-known inflammasome-forming receptor (Chaurasia et al., 2018). Besides these, microglia are known to express other inflammatory molecules, such as nitric oxide (NO) (Sierra et al., 2014) and reactive oxygen species (ROS) (Wang et al., 2014).

These glial cells work together to maintain retinal homeostasis, but under stress, they become activated, adapt their functions, and orchestrate inflammatory responses, as described in the next section.

## Transition from Homeostasis to Neuroinflammation in Glaucoma

### Triggers of glial activation

Even in their “resting state,” the glial cells are dynamically responding to the environmental cues while serving their principal homeostatic, metabolic, and immune functions, supporting cells of the retina (Fletcher et al., 2008). Gliosis has now been recognized as an active process that can be neuroprotective or deleterious depending on its context, magnitude, and duration. Therefore, in the following section, we discuss primary triggers of retinal gliosis, including oxidative stress, metabolic dysfunction, aging, and systemic inflammation.

#### Oxidative stress and metabolic dysfunction

The human retina is a highly energy-demanding tissue; therefore, disruption of mitochondrial electron transport enhances free radical production, and insufficient antioxidant defenses culminate in oxidative stress. Under physiological conditions, ROS are tightly regulated (Nebbioso et al., 2022); however, in diseases, such as glaucoma, DR, central retinal artery occlusion, and AMD, excess ROS overwhelms these defenses, causing oxidative damage and glial activation (Wang et al., 2022). Gliosis could be triggered by protein oxidation induced by the release of peroxiredoxin 2, hypoxia-inducible factor-1α, or proinflammatory cytokines (e.g., TNF-α, IL-1β) (B Domènech and Marfany, 2020). Oxidative stress also disrupts the glycocalyx, exposing photoreceptors and other cells to microglial phagocytosis and neuronal injury (Rashid et al., 2019). Excess ROS impairs glutamate cycling by reducing glutamine synthetase activity and glutamate uptake, leading to excitotoxicity, calcium-mediated mitochondrial damage, and a self-amplifying cycle of ROS production and RGC loss (Chrysostomou et al., 2013). MG initially release trophic and antioxidant factors, but chronic activation promotes neurotoxicity (Subirada et al., 2018). In the retina, ROS promote the expression of vascular endothelial growth factor (VEGF)A, driving neovascularization and capillary loss. Nicotinamide adenine dinucleotide phosphate oxidases (NOX) are major ROS sources: NOX2 drives DR and ischemia-reperfusion injury, microglia amplify ROS via NOX2/NOX4, NOX1/NOX4 promotes vascular leakage, and NOX5 (human-specific) exacerbates vascular damage (Drummond and Sobey, 2014; Ruan et al., 2020). Mitochondrial ROS further activate PARP and p38 mitogen-activated protein kinase (MAPK), promoting VEGF upregulation, endothelial dysfunction, and apoptosis (Zheng et al., 2010). eNOS uncoupling due to BH4 deficiency generates superoxide and peroxynitrite, impairing NO signaling (Ruan et al., 2020). In glaucoma, elevated IOP causes persistent oxidative stress in the retina and optic nerve, NOX2 upregulation, and endothelial dysfunction in retinal arterioles. Advanced glycation end-products (AGEs) and their receptor RAGE are elevated in RGCs and glia (Tezel et al., 2007), activating MAPK/nuclear factor kappa-B (NF-κB) signaling, ROS generation, inflammation, and apoptosis. Hyperlipidemia and apolipoprotein E-related dyslipidemia may exacerbate oxidative stress and vascular dysfunction, further increasing glaucoma risk. Together, ROS-driven neuronal and vascular dysfunction, compounded by glial dysregulation, underlie glaucoma.

Metabolic dysregulation represents another key pathological axis in glaucoma as it further amplifies glial activation. The retina relies predominantly on glucose, delivered via glucose transporters on retinal pigment epithelium (RPE), RGCs, and MG, for adenosine triphosphate (ATP) production through glycolysis and oxidative phosphorylation. MG spanning the full thickness of the retina, and astrocytes provide critical metabolic support to RGCs through glycogen storage, lactate shuttling (Magistretti, 2009), lipid transfer, potassium buffering, and glutamate clearance via excitatory amino acid transporter 1 transporters. Lactate produced through aerobic glycolysis in MG can be transferred to neurons as an alternative energy source, while glutamate is recycled into glutamine to prevent excitotoxicity (Rombaut et al., 2023). Microglia contribute to the clearance of debris and the release of trophic factors. In DR, chronic hyperglycemia disrupts these processes, causing mitochondrial dysfunction, energy depletion, ROS accumulation, and AGE formation (Viegas and Neuhauss, 2021; Chen et al., 2022).

In glaucoma, glia undergoes inflammatory and metabolic reprogramming. Initial stages show adenosine monophosphate-activated protein kinase activation in astrocytes and RGCs, with ONH astrocytes increasing glycolysis, glutamine use, and metabolic flexibility. Astrocytic glycogen, redistributed via connexin 43 gap junctions, can supply lactate to stressed regions but depletes donor reserves (Wender et al., 2000; Cooper et al., 2020). Microglia display early transcriptional changes in oxidative phosphorylation, glycolysis, and lipid metabolism, with glucose transporter upregulation and monocarboxylate transporters (MCT) (Tribble et al., 2020; Jassim et al., 2022). In later stages, loss of MCTs impairs lactate and pyruvate shuttling between glia and axons (Harun-Or-Rashid and Inman, 2018; Harun-Or-Rashid et al., 2020). Mitochondrial dysfunction in MG lowers ATP production, glutamate uptake (excitatory amino acid transporter 1), and potassium buffering (Kir4.1), exacerbating excitotoxicity (Djukic et al., 2007; Vohra et al., 2017). Oxidative stress markers (ROS, glutathione depletion, and hypoxia) are consistently elevated in glia after IOP elevation (Jassim and Inman, 2019). These metabolic disturbances weaken glial support to RGCs and may actively contribute to neurodegeneration.

#### Aging and systemic inflammatory signals

Aging is a significant risk factor for many neurodegenerative diseases, including glaucoma and AMD. It is associated with inflammaging, defined as the development of systemic chronic low-grade inflammation in the absence of infection (Chen et al., 2019). Aging has been associated with an increase in activated peripheral immune cells and proinflammatory cytokines. This, in turn, causes a decline in NAD^+^ levels, which may trigger inflammation and consequently affect retinal responses in degenerative conditions (Jadeja et al., 2020). Further, senescent cells can produce inflammatory cytokines during cellular senescence and senescence-associated secretory phenotype (Xu and Chen, 2022). Oxidative stress has also been known to trigger parainflammation, low-grade chronic inflammation (Xu et al., 2009). Cumulative oxidative stress can surpass homeostatic thresholds, activate glial cells, and contribute to neuronal dysfunction in age-related diseases (Kang et al., 2021). Key age-related alterations include astrocyte loss, oxidative stress in MG, and endothelial and neuronal senescence.

Aging disrupts the integrity and function of the inner BRB, primarily through changes in the neurovascular unit. BRB is fundamental to retinal homeostasis, functioning as a highly regulated and selective interface that meticulously controls the movement of ions, proteins, and water into and out of the retina. Structural deterioration of the neurovascular unit, including basement membrane thickening, pericyte abnormalities, and vascular changes such as capillary loss and leakage, has been documented in aging retinas (Roy and Kim, 2021; D’Esposito et al., 2025). Despite these changes, there is no evidence of T or B cell infiltration in normal aging. However, mild microglial and complement activation may occur, likely due to oxidative stress or compromised barrier function (Chen et al., 2019). Perhaps additional inflammatory signals, as in experimental autoimmune uveoretinitis (Chan et al., 1985) are required for immune infiltration. Activated microglia and MG contribute to BRB dysfunction during aging by releasing pro-inflammatory cytokines, promoting VEGF production, and disrupting tight junction proteins, thereby creating a self-perpetuating cycle of neuroinflammation (Fu et al., 2023). These glial-mediated processes, combined with hypoxia and impaired transcytosis, accelerate paracellular leakage, retinal edema, and susceptibility to vascular and degenerative retinal diseases (Hang et al., 2025; Zhang et al., 2025a). Furthermore, this may allow systemic inflammatory mediators, such as TNF-α, IL-6, and C-reactive protein, to reach the retina and activate glial cells. The above-discussed trigger and chronic exposure to these systemic cues contribute to ongoing gliosis and may prime the retina for accelerated degeneration in age-related diseases.

### Molecular pathways involved in neuroinflammation

Resident glial cells initiate conserved signaling cascades upon sensing the above-discussed triggers. Pattern recognition receptors, including Toll-like receptors (TLRs) and NOD-like receptors, detect both damage-associated molecular patterns (DAMPs) and pathogen-associated molecular patterns (PAMPs). In human glaucoma samples, TLR3 and TLR4 have been implicated in detecting DAMPs, like heat shock protein 27, heat shock protein 70, ATP, high-mobility group protein 1, and tenascin-C (Shestopalov et al., 2021; Salkar et al., 2024). TLR engagement activates downstream signaling pathways, including the MAPK, NF-κB, and Janus kinase/signal transducer and activator of transcription (JAK/STAT) pathways. These pathways drive the transcription of pro-inflammatory genes such as TNF-α, IL-1β, and IL-6, which are frequently elevated in retinal degenerative diseases (Salkar et al., 2024). The MAPK pathway, comprising extracellular signal-regulated kinase (ERK), JNK, and p38 kinases, also responds to pattern recognition receptors and cytokine signaling to regulate cellular stress, apoptosis, and cytokine production. Activation of p38 pathway was observed in microglia during early stages of glaucoma, influencing pro-inflammatory responses in microglia (Yu et al., 2021). In contrast, ERK affects trabecular meshwork contractility by negatively regulating Rho-kinase (Li et al., 2022). There was also widespread activation of ERK in the retina of glaucoma patients, prominently in activated microglia and astrocytes (Tezel et al., 2003). JNK isoforms exert complex, context-dependent effects. JNK2 and JNK3 mediate RGC death after acute injury; however, in ocular hypertension models, their absence does not prevent neurodegeneration, suggesting a role for JNK1. Notably, JNK2 may have a neuroprotective effect early in glaucoma, indicating that broad JNK inhibition requires caution (Harder et al., 2018). Selective NF-κB inhibition in glial cells helped in RGC survival (Lupien et al., 2013). Similarly, the JAK/STAT pathway activation in response to cytokines and interferons leads to the activation of both resident glial cells and infiltrating immune cells. Transient receptor potential vanilloid 4 (TRPV4) activation induced MG gliosis activates the JAK2/STAT3/NF-κB pathway, causing TNF-α release that leads to RGC death in glaucoma. While mammalian MG have limited regenerative ability, pathways like JAK/STAT and MAPK can enhance their proliferation and neurogenic potential (Beach et al., 2017; Li et al., 2021).

Cytokines play a crucial role in controlling the inflammatory milieu, as they determine whether the response resolves or progresses toward chronic inflammation and neurodegeneration. In the retina, IL-6, TNF-α, and IL-1β are produced by RGCs, activated microglia, astrocytes, and RPE cells. These cytokines disrupt retinal homeostasis as they modulate synaptic function, promote microglial activation, induce oxidative stress, and amplify cytokine release. Meanwhile, counter-regulatory cytokines, such as IL-10 and TGF-β, dampen excessive inflammation and promote cell survival (Russo et al., 2016). Activated microglia in glaucoma release TNF-α, IL-1β, and IL-6, which contribute to RGC death, tissue damage, and, paradoxically, neuroprotection against pressure-induced stress at least initially (Adornetto et al., 2019; Lin and Li, 2025). In addition to cytokine signaling, dysregulation of the complement cascade contributes significantly to retinal pathology, particularly in AMD. Complement activation increases with AMD progression, especially in patients carrying high-risk complement factor H and complement factor B variants (Heesterbeek et al., 2020). Overactivation of C3 and C5 generates anaphylatoxins (C3a, and C5a) that recruit immune cells, while formation of the membrane attacks complex damages retinal cells. Glaucoma progression is associated with upregulation of complement activators (C1Q, C3, and C7–9) and receptors, and downregulation of regulators, such as complement factor H and C4BP, notably in the inner retina and RGC layers (Hoppe and Gregory-Ksander, 2024). Elevated C3a/C3 ratios in aqueous humor (AH) and serum correlate with disease progression, highlighting complement activation as both a pathogenic factor and potential biomarker. The NLRP3 inflammasome is another critical inflammatory pathway, especially in microglia and RPE cells. Upon activation, often by oxidative stress or mitochondrial dysfunction, NLRP3 promotes caspase-1 activation, leading to the maturation and secretion of IL-1β and IL-18, both of which intensify tissue injury. This pathway is particularly implicated in AMD and DR (Doyle et al., 2012; Chaurasia et al., 2018). In glaucoma models, NLRP3 activation occurs early in the ONH following IOP elevation, preceding retinal changes, and is essential for RGC death (Puyang et al., 2016). This activation coincides with an increase in the production of pro-inflammatory cytokines and the infiltration of calcium-binding adapter molecule 1-positive microglia/immune cells. In summary, IOP-induced “danger” signals activate the NLRP3 pathway in ONH and retinal glia, leading to neurotoxic inflammation, axonal degeneration, and subsequently RGC loss (Gregory-Ksander et al., 2017; Coyle et al., 2021).

**Figure 1:**
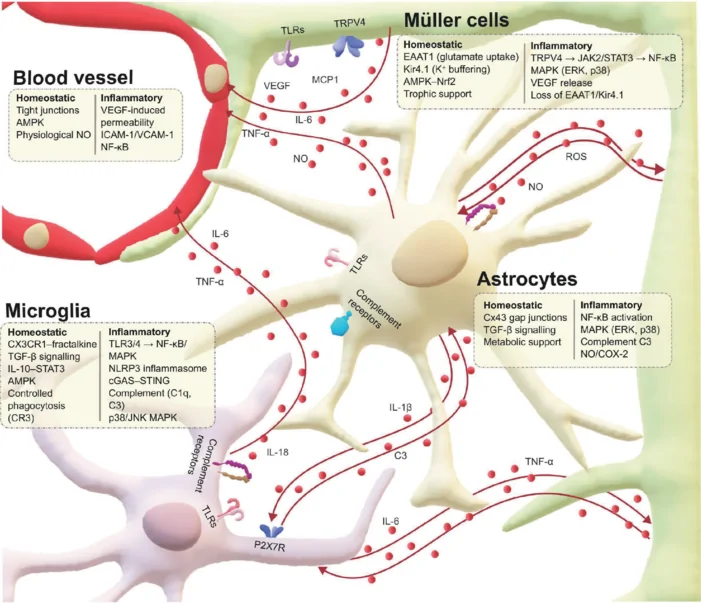
Pathways involved in inflammatory responses in the retina. The schematic depicts the roles of Müller gila, astrocytes, and microglia in maintaining retinal homeostasis and their involvement in neuroinflammation and BRB dysfunction. Inflammatory responses are typically triggered by oxidative stress, mitochondrial stress, mechanical stress (e.g., elevated IOP), excitotoxicity, or immune effectors. These stressors prompt affected cells, such as RGCs in glaucoma, to release damage signals. Glial cells expressing TLRs or other immune receptors detect these signals and become activated, initiating a multi-modal response. The schematic illustrates the pathways active in glial cells under both homeostatic and inflammatory conditions, with arrows highlighting the bidirectional flow of immune effectors. Together, these pathways drive a glial-mediated response that can amplify neuroinflammation. Created with Microsoft Paint 3D (Version 6.2410.13017.0). Akt: Protein kinase B; AMPK: AMP-activated protein kinase; ATP: adenosine triphosphate; BDNF: brain-derived neurotrophic factor; cGAS-STING: cyclic guanosine monophosphate–adenosine monophosphate synthase–stimulator of interferon genes; BRB: blood–retinal barrier; COX-2: cyclooxygenase-2; CNTF: ciliary neurotrophic factor; EAAT1: excitatory amino acid transporter 1; ERK: extracellular signal-regulated kinase; HMGP1: high mobility group protein 1; HSP: heat shock protein; ICAM1: intercellular adhesion molecule 1; IL: interleukin; IOP: intraocular pressure; JAK2: Janus kinase 2; JNK: c-Jun N-terminal kinase; MAPK: mitogen-activated protein kinase; NF-κB: nuclear factor kappa B; NO: nitric oxide; PI3K: phosphoinositide 3-kinase; RGC: retinal ganglion cell; ROS: reactive oxygen species; STAT3: signal transducer and activator of transcription 3; TGF-β: transforming growth factor-β; TLR: Toll-like receptor; TNF-α: tumor necrosis factor alpha; TRPV4: transient receptor potential vanilloid 4.

The cyclic guanosine monophosphate–adenosine monophosphate synthase-stimulator of interferon genes (cGAS–STING) pathway has also been highlighted as a contributor to chronic retinal inflammation. This pathway is activated in response to cytosolic DNA, leaked from mitochondria or the nucleus, during retinal stress, resulting in persistent type I interferon signaling and sustained immune activation. In animal models of glaucoma, activation of microglial cGAS–STING signaling occurred early in the disease timeline and was detrimental to RGC survival (Liu et al., 2024). Together, these pathways orchestrate protective immune responses; however, when dysregulated, they result in chronic inflammation, sustained glial activation, and progressive retinal degeneration. A schematic representation of the pathways involved in inflammation is provided in **Figure 1**.

### Morphological and functional diversity in homeostasis and activation

**Figure 2:**
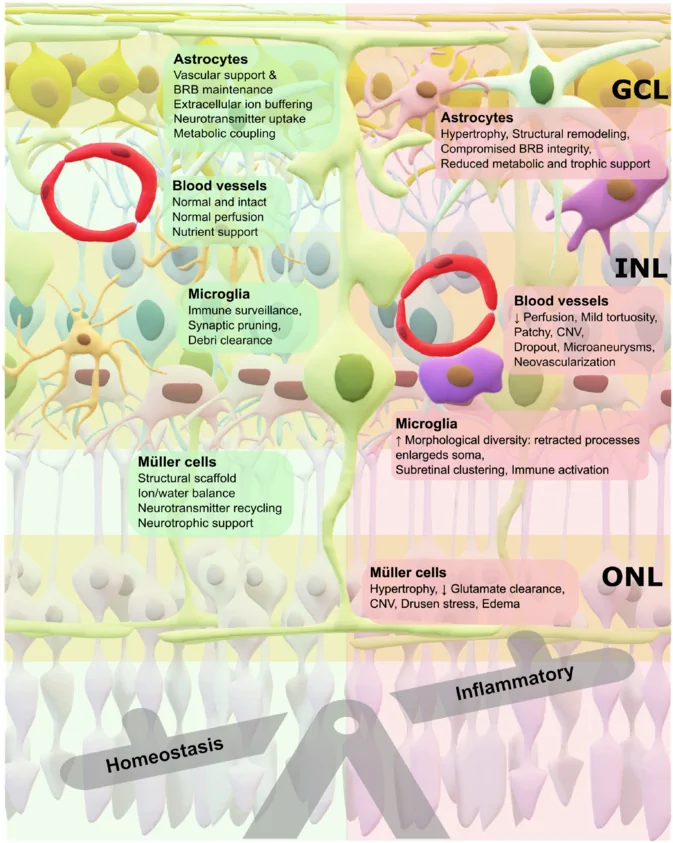
Retinal glial cell types, their roles under homeostatic and inflammatory conditions. Retinal glial cells, including Müller cells, astrocytes, and microglia, play a unique role in maintaining retinal homeostasis and structural organization. Under physiological conditions, they are characterized by functional specializations that support neuronal survival and metabolic balance. When exposed to stress signals or injury, these cells become activated and may shift towards either neuroprotective or pathogenic responses, depending on environmental cues. This transition is characterized by altered marker expression, changes in signaling pathways, and distinct phenotypic and morphological adaptations that influence the progression of retinal disease. Created with Microsoft Paint 3D (Version 6.2410.13017.0). BRB: Blood–retinal barrier; CNV: choroidal neovascularization; GCL: ganglion cell layer; GS: glutamine synthetase; INL: inner nuclear layer; ONL: outer nuclear layer.

Activation of the immune pathways can lead to functional and morphological changes in the glial cells, ranging from protective adaptations to maladaptive states that contribute to neurodegeneration, as illustrated in **Figure 2**. This section describes the morphology and phenotypic transitions of each glial subtype in both homeostatic and reactive states.

#### Microglia

Microglia are the retina’s immune sentinels, responding rapidly to injury, stress, or infection (Murenu et al., 2022). In homeostasis, they display a ramified morphology with fine, motile processes, maintaining immune surveillance, synaptic integrity, neurotrophic support, debris clearance, and crosstalk with neurons and glia. Upon activation, microglia retract processes, enlarge their soma, increase phagocytic capacity, and upregulate immune mediators (TNF-α, IL-1β, and IL-6) and markers (cluster of differentiation 68 (CD68), human leukocyte antigen – DR isotype (HLA-DR), and ionized calcium-binding adapter molecule 1) (Colonna and Butovsky, 2017). Previously categorized as M1 (pro-inflammatory) or M2 (anti-inflammatory), microglia are now recognized to exist along a continuum, modulated by local cues and inhibitory neuron–microglia pathways, such as CD200-CD200R and CX3CL1-C–X3–C chemokine receptor 1 (Murenu et al., 2022). Morphological states include hypertrophic/hyper-ramified (early gliosis), bushy/dystrophic (aging or chronic degeneration), rod-shaped (aligned with degenerating axons), and satellite (adjacent to neurons or glia) (Savage et al., 2019). High-throughput studies have identified specialized subtypes, including disease-associated microglia, proliferative-region-associated microglia, axon tract-associated microglia, and CD11c-positive microglia (Rosmus and Wieghofer, 2022). Studies in the mouse retina have shown that microglial activation and morphological transformations are regulated by interactions with other retinal cells and neurotransmitters (Fontainhas et al., 2011).

Human post-mortem studies in glaucoma report amoeboid microglial clusters at the lamina cribrosa, especially around blood vessels, expressing HLA-DR, CD68, TNF-α, NO synthase 2 (NOS2), COX-1, proliferating cell nuclear antigen, and metalloproteases, reflecting proliferative and activated states (Yuan and Neufeld, 2001). Early activation balances pro- and anti-inflammatory states, but hyperactivation amplifies inflammatory signaling, releases neurotoxic mediators, and accelerates RGC degeneration. These studies have also reported both amoeboid and intermediate microglial morphologies (Rutigliani et al., 2022; Salkar et al., 2025). Activated microglia exhibits spatially heterogeneous marker expression (CD45, CD11b, HLA-DR, transmembrane protein 119 (TMEM119), and CD68). Therefore, supporting a continuum model and underscoring the importance of characterizing microglial states for neuroprotection.

#### Müller glia

MG are radially oriented cells that span the entire thickness of the retina and provide the homeostatic and metabolic support of retinal neurons. They display topographical heterogeneity, with type I cells showing numerous thin processes and type II cells fewer, thicker processes in the peripheral retina (Anezary et al., 2001). This heterogeneity can be extended to regional differences in protein expression, such as higher phosphoglycerate dehydrogenase (Zhang et al., 2019) and aquaporin-4 (Daruich et al., 2018) in macular MG, indicating variable susceptibility to oxidative stress and disease. Peripheral MG retain regenerative potential, expressing CD44 and nestin post-injury, and respond differently to elevated IOP, influencing regional RGC vulnerability. Animal glaucoma models show peripheral MG proliferate more, express higher stem cell and Wnt/β-catenin markers, and secrete neuroprotective factors, yet are more susceptible to hydrostatic stress, upregulating TRPV4/Piezo1, pro-apoptotic, inflammatory, and oxidative stress proteins, reducing RGC survival (Pereiro et al., 2024). MG involvement in glaucoma pathogenesis is also thought to occur via dysregulated purinergic and glutamatergic signaling. Elevated IOP stimulates ATP release from MG, activating autocrine P2Y receptors and RGC P2X7 receptors, causing Ca^2+^ overload and RGC death, while impaired P2Y6 signaling compromises neurite maintenance. Reduced Kir4.1 channel function diminishes glutamate uptake, elevates extracellular K^+^, and triggers excitotoxic N-methyl-D-aspartate/α-amino-3-hydroxy-5-methyl-4-isoxazolepropionic acid receptor activation, exacerbating degeneration (Shinozaki and Koizumi, 2021). MG heterogeneity, shaped by retinal topography, molecular expression, and mechanosensory responses, influences both neuroprotection and vulnerability, highlighting the need for region- and cell state-specific therapeutic strategies.

#### Astrocytes

Retinal astrocytes are the most prominent cell type in the non-myelinated ONH (Cullen and Sun, 2023). They are known to exhibit two morphologies: elongated (support the axons in nerve fiber layer) and stellate (honeycomb-shaped network in the ganglion cell layer). Functional heterogeneity remains poorly characterized; single-cell RNA sequencing studies have not clearly distinguished subtypes (Lukowski et al., 2019). Post-mortem glaucoma studies report increased GFAP intensity, particularly peripapillary, and elevated connexin 43, indicating enhanced gap-junction communication (Kerr et al., 2011). In addition, there was no correlation between astrocyte activation and glaucoma severity (Wang et al., 2002). Animal models similarly reveal stage- and region-dependent astrocyte redistribution (Formichella et al., 2014).

ONH astrocytes support axons, maintain energy metabolism, and ameliorate ROS. In addition, these astrocytes have been known to respond to mechanical pressure; therefore, they could potentially be involved in glaucoma pathology (Choi et al., 2015). However, the changes to the astrocyte functions have been associated with RGC axon loss (Bosco et al., 2016). They contribute to extracellular matrix (ECM) remodeling to preserve structural integrity (Hernandez, 2000; McGrady et al., 2021) and maintain ion exchange and energy transport in myelinated regions via gap junctions (Wender et al., 2000).

Astrocytes also actively contribute to immune responses within the retina and optic nerve. They contribute to neuroinflammation by expressing NOS and TNF-α, which are upregulated in glaucoma and may directly damage RGCs. Astrocytic response is closely tied to the A1/A2 astrocyte paradigm: A1 astrocytes, typically induced by microglia-derived IL-1α, TNF-α, and C1q, acquire a neurotoxic phenotype that contributes to synaptic loss and neuronal death. In contrast, A2 astrocytes exhibit a neuroprotective profile, promoting survival and regeneration through the secretion of growth factors and anti-inflammatory mediators. Although this binary classification is likely oversimplified, it describes the context-dependent plasticity of astrocyte responses (Liddelow and Barres, 2017; Tang et al., 2022). However, information regarding the distribution of astrocyte subtypes and their association with disease progression remains limited and poorly understood.

Emerging evidence suggests that astrogliosis not only involves proliferation but also redistribution and increased functional activity of astrocytes, aimed at preserving axon integrity even during degeneration (Cooper and Calkins, 2024). Highlighting the duality of astrocyte responses in disease: while they may initially offer neuroprotection, prolonged activation or maladaptive responses can exacerbate damage. Further, astrocyte interactions with other glia (e.g., microglia and MG), as well as components of the neurovascular unit, remain underexplored in glaucoma and may hold key insights into disease progression.

## Glial Cell Dysfunction in Other Retinal Diseases

Chronic retinal diseases, such as DR, AMD and retinal vascular occlusions, share key inflammatory mechanisms with glaucoma. Yet, they differ in etiology, primary affected cell types, and patterns of glial involvement. DR arises from hyperglycemia-induced metabolic stress that drives vascular and neuroinflammatory changes across the retina, including MG hyperplasia, astrocyte loss, and microglial hypertrophy (Grigsby et al., 2014). In AMD, chronic oxidative stress and complement dysregulation at the RPE led to microglial and macrophage activation, drusen accumulation, and photoreceptor loss (Whitcup et al., 2013). In retinal vascular occlusions, ischemia-driven hypoxia and reperfusion injury lead to endothelial damage and inflammatory signaling, which in turn trigger microglial activation, Müller glial reactivity, vascular leakage, and regional neuronal loss (Zhang et al., 2025b). Although all three diseases involve glial activation and inflammatory signaling, the spatial localization, morphological adaptations, and primary triggers of gliosis differ, reflecting disease-specific mechanisms that converge on retinal degeneration. The following section provides a brief overview of glial cells and immune responses in AMD, DR and retinal vascular occlusions, summarized in **Additional Table 1**.

### Diabetic retinopathy

DR is classified based on ophthalmoscopically visible vascular and related lesions. It progresses from a non-proliferative stage, marked by vascular tortuosity, retinal hemorrhages, microaneurysms, and lipid exudates, to a proliferative stage, characterized by the growth of fragile, aberrant neovessels. Among diabetes-related microvascular complications, including nephropathy and neuropathy, DR is the most prevalent. Its pathogenesis involves multiple complex inflammatory mechanisms. Systemic low- or high-grade inflammation drives structural and molecular alterations in the neurovascular unit; however, the precise inflammatory pathways involved remain incompletely understood. Pathological microvascular changes in DR include dilated veins, microaneurysms, hemorrhages, cotton-wool spots, and vitreoretinal neovascularization (Yu et al., 2015).

In DR, MG exhibit hyperplasia as early as 12 weeks, with ultrastructural changes including chromatin dispersion, glycogen accumulation, and swollen endfeet preceding increased GFAP expression (Yang et al., 2022). MG respond to microenvironmental cues and act as significant sources of inflammatory mediators, such as VEGF, IL-1β, TNF-α, monocyte chemoattractant protein-1, and intercellular adhesion molecule-1 (Gerhardinger et al., 2005), regulated via pathways such as Wnt/β-catenin, p38 MAPK/NF-κB, and RAGE–MAPK (Zong et al., 2010; Zhou et al., 2014). Hyperglycemia further induces the production of NO and prostaglandin E₂ via iNOS and COX-2. IL-1β activation through caspase-1 contributes directly to DR pathology (Yu et al., 2015). Astrocytes in DR show reduced density, particularly in the peripapillary and peripheral retina, with retraction of processes from axonal bundles (Yu et al., 2015). While astrocytes can exert anti-angiogenic effects via exosomal cargo, they also engage in pro-inflammatory cascades. Induction of COX-2 promotes prostaglandin E_2_-driven VEGF expression, while TGF-α–mediated epidermal growth factor receptor activation amplifies COX-2 responses after injury. Hyperglycemia and IL-1β contribute to the sustained activation of astrocytes, thereby promoting chronic inflammation (Zhang and Neufeld, 2005; Liu et al., 2012a). Microglia in DR become hypertrophic, cluster around damaged vessels, and infiltrate both the optic nerve and retinal parenchyma (Yu et al., 2015). Activated microglia release neurotoxic and pro-angiogenic factors, including TNF-α, IL-1β, IL-6, VEGF, glutamate, MMPs, leukotrienes, and chemokines (Grigsby et al., 2014). Hyperglycemia stimulates purinergic P2 receptor upregulation, driving Ca^2+^ influx and cytokine release (Pereira Tde et al., 2010). NF-κB activation is critical for pathological angiogenesis, and its inhibition mitigates neuronal loss (Yoshida et al., 1999). Pharmacological suppression of microglia with agents, such as minocycline (Krady et al., 2005) or pigment epithelium-derived factor-derived peptides (Liu et al., 2012b) reduces cytokine production, caspase-3 activation, and retinal thinning.

### Age-related macular degeneration

AMD remains a significant cause of severe visual impairment worldwide and is defined by the degeneration of macular photoreceptors and RPE, together with the accumulation of subretinal deposits containing oxidized lipids and proteins, termed drusen (Dhodapkar et al., 2022). Clinically, early AMD presents with soft drusen and/or pigmentary abnormalities. In its advanced stages, the disease manifests as either geographic atrophy (GA, and dry AMD), characterized by photoreceptor and RPE cell loss, or as the exudative (wet) form, in which abnormal angiogenesis gives rise to a choroidal neovascular (CNV) membrane (Nita et al., 2014). Multiple pathogenic drivers, including oxidative stress, aging, DNA damage, and ultraviolet radiation, converge to impair RPE autophagy, promote cellular senescence, and activate immune-inflammatory pathways, all of which are interconnected and mutually reinforcing. Chronic inflammation is a key feature of AMD and other age-related retinal diseases, with progression linked to dysregulated immune responses that compromise protective barriers, such as the BRB and the eye’s intrinsic anti-inflammatory mechanisms (Wang et al., 2019).

Drusen, which accumulate between the RPE and choroid, represent a compartmentalized aggregation of cellular debris enriched with oxidized lipids and proteins of RPE origin, complement components, amyloid-β peptide, serum amyloid P, immunoglobulin light chains, and vitronectin (Crabb et al., 2002). Doyle et al. (2012) demonstrated that internalization of drusen constituents can activate the NLRP3 inflammasome in human RPE, triggering secretion of the pro-inflammatory cytokines IL-1β and IL-18 via NF-κB pathway. Complement-related proteins have been localized not only to drusen, but also to choroidal capillary pillars, excised CNV membranes, and the vitreous body. Complement activation serves as both a marker and a driver of chronic, localized immune dysregulation at Bruch’s membrane, with dysregulation implicated in both dry and wet AMD (Nita et al., 2014).

Retinal immune cells, including microglia, macrophages, and dendritic cells, are also activated in AMD, producing a broad spectrum of inflammatory mediators. Macrophage polarization plays a significant role: M1 pro-inflammatory macrophages exacerbate retinal injury, whereas M2 macrophages may aid in tissue repair. Experimental models show an accumulation of innate immune cells within the subretinal space, and human AMD lesions, whether drusen or GA, contain immune cell infiltrates (Fletcher, 2020). Infiltrating microglia can impair RPE function through positive feedback interactions, further undermining retinal immune privilege and contributing to AMD pathology (Lin et al., 2023). Cytokines, such as IL-17, produced by T cells and innate lymphoid cells, have been shown to promote intraocular neovascularization and photoreceptor injury in AMD (Nita et al., 2014; Ascunce et al., 2023). Thus, AMD reflects immune privilege breakdown, inflammasome–complement crosstalk, and persistent glial/immune activation, though direct mechanistic insights into glia remain limited.

### Retinal vascular occlusions

Retinal vascular occlusions (retinal vein occlusions (RVOs) and retinal arterial occlusions (RAOs)) are significant causes of vision loss, with distinct mechanisms despite overlapping systemic risk factors, including hypertension, diabetes, dyslipidemia, and smoking. RAOs, including central retinal artery occlusion and branch RAO, represent acute ischemic events most often caused by emboli or thrombosis from carotid atherosclerosis or cardiac sources, leading to irreversible retinal damage within hours and poor visual prognosis, with less than 20% of central retinal artery occlusion patients regaining useful vision. In contrast, RVOs, central RVO, hemi-RVO, and branch RVO, arise from venous stasis, vessel wall degeneration, and hypercoagulability (Virchow’s triad), often at arteriovenous crossings. Their complications, including macular edema, ischemic maculopathy, and neovascularization, are now effectively managed with anti-VEGF therapies. Together, RAOs reflect acute arterial ischemia with limited treatment options, whereas RVOs exemplify venous insufficiency with more tractable therapeutic avenues (Srejovic et al., 2024).

In retinal vascular occlusions, ischemia could rapidly disrupt retinal energy metabolism, leading to ATP depletion, excitotoxicity, calcium overload, and neuronal death. This would trigger the downstream cascade, wherein hypoxia stabilizes hypoxia-inducible factor-1α, which in turn induces VEGF, angiopoietins, and platelet-derived growth factor (Zhou et al., 2023). This drives neovascularization, vascular leakage, and BRB breakdown. In RVO, VEGF overproduction has been strongly associated with macular oedema and ischemic angiogenesis, whereas in RAO, it has been known to cause abrupt ischemia, triggering an acute oxidative burst that accelerates ganglion cell degeneration. Inflammatory cascades further exacerbate vascular injury. TNF-α, IL-1β, and IL-6 increase endothelial permeability and apoptosis. NF-κB and TLR signaling promote leukocyte adhesion, fibrosis, and chronic inflammation. Activated microglia release cytokines and ROS that destabilize the vasculature (Jovanovic et al., 2020; Zhang et al., 2025b).

Experimental models of branch RVO induced by laser injury demonstrate increased microglial activation and macrophage infiltration. This response is marked by elevated levels of TNF-α, IL-1β, and IL-6, which disrupt vascular stability and exacerbate neuronal injury. Notably, microglial suppression improves RGC survival by dampening the innate immune response (Jovanovic et al., 2020). Activated microglia also interact closely with MG, which are essential for BRB integrity. Under ischemic stress, MG undergo reactive gliosis, releasing VEGF and other permeability-inducing mediators. Their swollen endfeet compromise endothelial tight junctions, further promoting vascular leakage and accelerating BRB breakdown (Zhang et al., 2025b). Another study indicated that this immune response is primarily driven by ocular-resident cells rather than circulating immune cells, with pro-inflammatory cytokine upregulation more prominent in central RVO than in branch RVO (Zhou et al., 2023). Together, hypoxia, VEGF signaling, inflammation, and oxidative stress form a self-perpetuating loop of neurovascular injury underlying the pathophysiology of occlusive retinal disease.

Although numerous studies have investigated the role of glial cells in retinal disease, much remains to be explored before the underlying disease mechanisms are fully understood. Platforms that enable advanced disease modeling and high-throughput multi-omics analyses offer critical opportunities to address these gaps and are reviewed and critically evaluated in the following sections.

## Humanized Models and Biomarkers for Better Navigation of Glial Modulation and Disease Prognosis

### Translational limitations of rodent models

Experimental animal models have played a central role in understanding glaucoma, encompassing both small (rodents) and large (non-human primates) species (Agarwal and Agarwal, 2017). While large animals provide anatomical fidelity, their use is limited by cost, ethics, and accessibility, making rodents the predominant choice. Glaucoma models typically involve direct RGC injury (e.g., optic nerve crush) or elevated IOP (e.g., microbead injection) (Evangelho et al., 2019; Tirendi et al., 2020). AMD models employ genetic mutants (complement, chemokine, lipid metabolism), environmental stressors (oxidative damage, diet, light), and injury paradigms (laser- or VEGF-induced CNV), each reproducing discrete disease features (Pennesi et al., 2012). DR relies heavily on rodents, through pharmacological (streptozotocin, alloxan), diet-induced, or genetic models (Ins2Akita, Leprdb, and GK), while larger animals and zebrafish provide complementary vascular insights (Quiroz and Yazdanyar, 2021). RVO and RAO models employ laser, chemical, or surgical induction across species, with rodents commonly used for practicality and larger animals offering greater anatomical similarity to humans (Khayat et al., 2017; Vestergaard et al., 2019). Although studies have incorporated glial cells through co-culturing (Wang et al., 2023) and/or repopulation (Cheng et al., 2023), these approaches often fail to capture the complexity of human diseases, which are inherently multimodal in nature. Despite their utility, these models often employ young animals and fail to recapitulate the chronic, multimodal, and age-related complexity of human disease. This limitation describes the need for humanized models that more accurately reflect disease heterogeneity, comorbidities, and progression over time.

### Humanized studies and disease model establishment

Humanized systems complement animal models by more accurately reflecting human retinal biology, particularly in terms of glial dynamics. Post-mortem human tissues offer high anatomical fidelity, enabling in situ mapping of microglial activation, astrocytic hypertrophy, and Müller gliosis, making them valuable for defining disease-specific glial phenotypes and regional heterogeneity. However, they represent static snapshots and are limited by donor availability. Comparative analysis showed disease-specific patterns in glial activation across AMD, DR, and glaucoma (Salkar et al., 2025). Also, glial activation was found to be independent of RGC loss in early and end-stage samples (Rutigliani et al., 2022). Further studies have also shown the presence of infiltrated immune cells in post-mortem retina (Margeta et al., 2018; Rutigliani et al., 2022; Salkar et al., 2024). Advancements in spatial transcriptomics and multi-omics enable high-resolution mapping of gene expression, protein localization, and metabolism, uncovering glial subtype–specific profiles and disease-driving pathways (Cheng et al., 2025), whilst retaining the architectural context of the tissue. Systems biology approaches such as imaging mass spectrometry (Desbenoit et al., 2013; Anderson et al., 2015) and spatial transcriptomics (Zhang et al., 2024b) have begun to yield important insights into the pathophysiological landscape of glaucoma and other retinal diseases (Cheng et al., 2025). However, current studies still fall short of distinguishing functional glial phenotypes, limiting the ability to link molecular signatures with disease mechanisms.

Organ culture systems bridge the gap between static human tissue and reductionist cell models by retaining retinal architecture and enabling physiologically relevant investigation of cell–cell interactions. Several methodologies have been employed in this context, including retinal slice preparations, explant cultures, and whole-eye organ cultures (Schnichels et al., 2019). Retinal slice preparations utilize transverse retinal sections that preserve local microglia–neuron and astrocyte–Müller interactions, enabling high-resolution studies of rapid glial signaling, but are largely restricted to rodent models and short-term experiments (Kulkarni et al., 2015; Franke et al., 2017). In contrast, human retinal explants and whole-eye cultures preserve tissue organization and allow investigation of intercellular signaling using post-mortem samples. Recent advances have extended their viability beyond the early 48-hour window, permitting analysis of progressive neuronal and glial degeneration (Niyadurupola et al., 2011; Osborne et al., 2016). These models have been instrumental in delineating early degenerative patterns in neuronal and glial populations, revealing progressive degeneration of retinal cells in human neuroretina cultures (Fernandez-Bueno et al., 2012). Choroidal explants have been leveraged to study angiogenesis and angiofibrosis to model AMD (Miller et al., 2025). Although still in early stages of application, these systems provide unique opportunities to assess glial cell activation and crosstalk longitudinally under pathological conditions.

Cell-culture-based systems, encompassing immortalized cell lines, primary cultures, organoids, and organ-on-a-chip platforms, have been employed to investigate glaucoma pathophysiology; however, these models frequently fail to recapitulate the full complexity of native retinal tissue. Glaucoma models employ elevated hydrostatic pressure (Yu et al., 2011; Aires et al., 2017) or oxidative and excitotoxic stress (Quill et al., 2011) to investigate mechanisms of RGC death, glial activation, and neuroinflammation. Co-culture systems comprising RGCs with glial cells, whether in direct contact or via Transwell configurations, provide a platform to investigate paracrine signaling, immune-mediated cytotoxicity, neuroprotective mechanisms, and the contributions of glial cells and the neurovascular unit to RGC survival (Wisniewska-Kruk et al., 2012; Green and Ou, 2015). Even with some studies leveraging human-derived cells to mimic the BRB (Fresta et al., 2020; Maurissen et al., 2024), animal-derived cells remain famous and extensively used. A recent study reported that co-culture and triculture models of human-derived pluripotent stem cells, including astrocytes, microglia, and RGCs (Harkin et al., 2025). This work provides pilot evidence for building co-culture or tri-culture models to understand the neuro-immune aspects of glaucoma and other retinal diseases.

Three-dimensional retinal organoids derived from human pluripotent stem cells provide a developmentally relevant, laminated retinal architecture (Ma et al., 2024). Organ-on-a-chip technologies, which are microfluidic systems that recreate key aspects of ocular physiology, such as fluid dynamics, pressure gradients, vascular flow, and immune infiltration, offer powerful tools for modeling IOP effects and complex cell–cell interactions (Maurissen et al., 2024). Together, these human-derived systems provide physiologically relevant and manipulable models for mechanistic studies and translational investigation of retinal diseases, such as glaucoma, AMD, and DR, with utility in dissecting the contributions of glia to retinal pathology. A persistent limitation across these platforms is the underrepresentation of retinal glia, as most models are designed to interrogate neuronal or vascular pathology, with glial alterations often treated as secondary readouts. To this end, we have highlighted the applications of all the above-discussed model systems to study glial cells in **Additional Table 2**.

**Additional Table 2:** Summary of humanized models for assessing glial cells in retinal diseases

Model type	Platform/approach	Key feature/capability	Advantage	Limitation	Application	Reference
Post-mortem human tissues	Retina, ONH, RPE, sclera	Enables mapping of microglial activation, astrocytic hypertrophy, and Müller gliosis in situ	High anatomical fidelity, Spatial resolution	Static snapshot; limited clinical metadata; rare availability	Defining disease-specific glial phenotypes and regional heterogeneity	Rutigliani et al., 2022; Cheng et al., 2025; Salkar et al., 2025
Retinal slice cultures	Transverse retinal sections	Preserve microglia-neuron and astrocyte-Müller interactions in local architecture	High spatial resolution: studies cell-cell interactions	Short viability (~4 h); mostly rodent models	Study rapid glial calcium signaling, gliotrans mission, and neuron-glia coupling	Kulkarni et al., 2015; Franke et al., 2017
Human retinal explants/whole-eye cultures	Explants cultured on inserts or plates	Maintain Müller-astrocyte scaffolding and microglial distribution	Physiologically relevant; human-derived Longer viability (up to 48+h)	Limited long-term culture; donor variability and availability	Probe glial responses to ischemia, excitotoxicity, and test neuroprotective interventions	Niyadurupola et al., 2011; Osborne et al., 2016
2D cell culture	Cell lines, primary cells,	Allow stress paradigms (oxidative, pressure, hypoxia) on isolated glia	Simple, reproducible, mechanistic studies	Lacks 3D architecture, cellular diversity, and gradient dynamics	Study reactive gliosis, cytokine/VEGF release, and signaling pathways	Aires et al., 2017
Co-culture systems	RGCs + astrocytes/Müller cells/micro glia	Model neuron-glia crosstalk and paracrine signaling	Model paracrine signaling and glial contributions	Often animal-derived, human cells are limited	Investigate glia-driven RGC survival, synapse remodeling, and neuroprotection	Wisniews ka-Kruk et al., 2012; Green and Ou, 2015; Harkin et al., 2025
Blood-retinal barrier models	Transwell or microfluidic setups	Endothelial-Müller-astrocyte interactions; leukocyte transmigration studies	Simulate retinal microenvironment	Mostly animal-derived; human-derived examples are limited	Understand glial contribution to barrier integrity and inflammatory responses	Fresta et al., 2020
3D retinal organoids	hPSC-derived retinal organoids	Develop Müller cells and astrocyte-like glia; partial microglial integration possible	Physiologically relevant; developmental context	Long-term RGC survival, vascular/immune integration, and age-related modelling are challenging	Model glial maturation, gliosis, and their role in retinal development or degeneration	Ma et al., 2024
Organ-on-a-chip	Microfluidic platforms	Incorporate glial responses to flow, pressure, and immune infiltration	Model IOP effects, biomechanical/biochemical cues	Complex fabrication; partial recapitul ation of the human retina	Model glaucoma-specific glial stress, vascular-glial crosstalk, and mechanobiology	Maurissen et al., 2024

2D: Two-dimensional; 3D: three-dimensional; BRB: blood-retinal barrier; hPSC: human pluripotent stem cell; IOP: intraocular pressure; Müller: Müller glia; ONH: optic nerve head; RGC: retinal ganglion cell; RPE: retinal pigment epithelium; Transwell: Transwell insert system; VEGF: vascular endothelial growth factor.

However, even with the advent of such studies, the development of robust glial-centered models, achieved through advanced organoid maturation, microfluidic glia–vascular platforms, or extended co-culture paradigms, remains an essential frontier for glaucoma research.

### Biomarker discovery from human retina and biofluids

Humanized animal models are useful for studying glaucoma pathophysiology, testing therapies, and validating drug candidates, but no model fully captures the complex, multifactorial nature of human disease. This highlights the importance of integrating human-derived samples, especially biofluids, to advance translational research. While retinal and optic nerve tissues provide direct pathological insights, they are largely inaccessible in living patients. AH and vitreous humor are informative but require invasive collection. In contrast, blood and tears are easily accessible and can reflect systemic or local changes, including inflammatory, metabolic, and autoantibody profiles. Advances in proteomics analysis are increasingly enabling the sensitive detection of these alterations, offering promise for non-invasive diagnostics, disease monitoring, and personalized therapeutic strategies.

**Figure 3:**
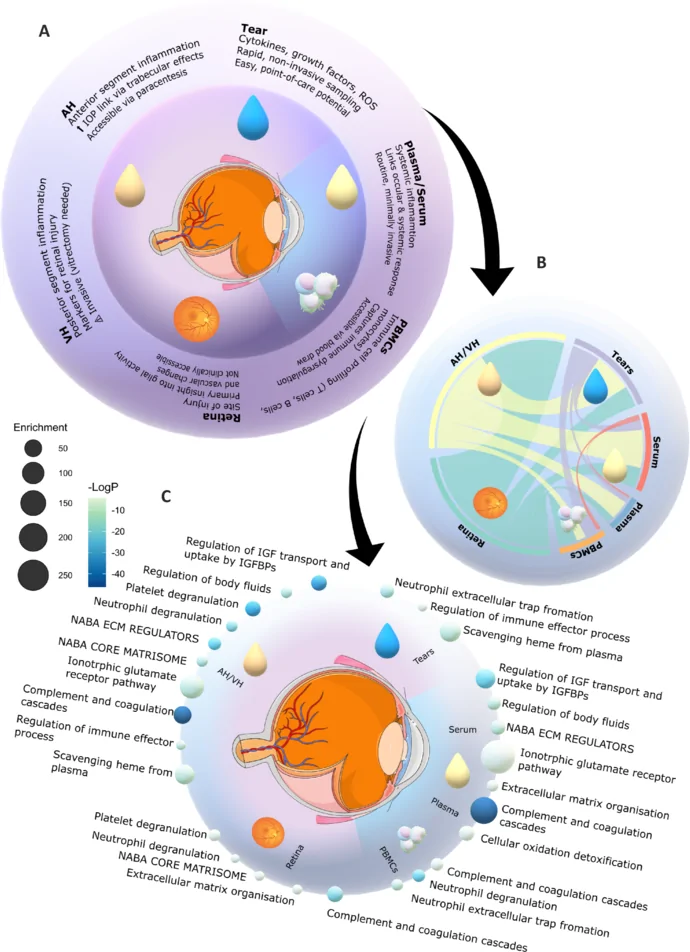
Proteomics workflow for biomarker discovery in retinal diseases. (A) Overview of the mass-spectrometry-based proteomics workflow used to investigate both local (retina, aqueous humor, and vitreous humor) and systemic (plasma/serum, and PBMCs) responses in retinal diseases. (B) Integrative data analysis from glaucoma studies. Circos plot shows shared differentially expressed proteins across tissues, with tissue-specific enriched pathways represented as dots with dot size representing enrichment score and color representing statistical power. (C) Overlaps across different proteomic platforms indicate that glaucoma has systemic effects beyond the eye, highlighting the need for multi-compartment studies. Vectors adapted from Servier Medical Art (https://smart.servier.com/), licensed under CC BY 4.0 (https://creativecommons.org/licenses/by/4.0/). AH: Aqueous humor; ECM: extracellular matrix; IGF: insulin-like growth factor; IGFBPs: insulin-like growth factor–binding proteins; NABA: matrisome classification framework defined by the NABA project; PBMCs: peripheral blood mononuclear cells; VH: vitreous humor.

In this section, we highlight the use of proteomics-based approaches to characterize alterations in the retina, ONH, AH, peripheral blood, and peripheral blood mononuclear cells in glaucoma patients (**Figure 3**). In proteomics studies, discovery-phase investigations often rely on label-free DDA and DIA methods to identify differentially expressed proteins, laying a foundation for biomarker discovery and a deeper understanding of glaucoma pathology. To enhance proteome coverage and reproducibility, advanced techniques such as high-resolution mass spectrometry and sequential window acquisition of all theoretical mass spectra have been adopted. For translational purposes, targeted quantification strategies, including multiple reaction monitoring, parallel reaction monitoring (PRM), and Olink, enable sensitive and specific validation of candidate biomarkers. High-throughput proteomics of fixed ocular tissues have revealed significant alterations associated with glaucoma. These studies have shown that energy metabolism impairment, mitochondrial dysfunction, and altered immune responses are common pathways affected in the glaucoma retinae even before neurodegeneration sets in. In the sclera, alterations were observed in ECM remodeling, cytoskeletal regulation, and protein glycosylation (Iomdina et al., 2020). Overall, proteomics studies using retinal, scleral, and ONH tissues revealed that energy dysregulation, oxidative stress, immune responses and ECM remodeling converge to drive RGC vulnerability and ONH pathology (Yang et al., 2015; Funke et al., 2016; Mirzaei et al., 2017; Iomdina et al., 2020).

AH has emerged as a valuable biofluid for proteomic biomarker discovery, owing to its proximity to affected ocular tissues and accessibility during surgery. AH proteomics consistently shows upregulation of metabolic, complement, and immune-related proteins with alterations to the barrier integrity and distinct molecular signatures across glaucoma subtypes (Izzotti et al., 2010; Anshu et al., 2011; Kaeslin et al., 2016; Salamanca et al., 2018; Mok et al., 2024). Even studies using targeted approaches, such as Olink (Zhang et al., 2024a) or multiple reaction monitoring (Kaur et al., 2019) confirmed significant upregulation of metabolic and complement-related proteins, reflecting immune dysregulation and oxidative stress. Tear proteomics has emerged as a promising, non-invasive approach for biomarker discovery; however, variability between samples remains a significant limitation. In treatment-naïve primary open-angle glaucoma patients, significant dysregulation of immune, inflammatory, and metabolic proteins has been reported (Pieragostino et al., 2013; Rossi et al., 2019). Inflammatory processes were also found to be dysregulated in patients undergoing chronic topical antiglaucoma therapy (Wong et al., 2011), suggesting that chronic treatment may induce a distinct, drug-related ocular surface inflammatory response. Lastly, Spörri et al. (2024) demonstrated enrichment of immune-related proteins despite minimal microbiome differences, underscoring the contribution of local immunological processes to disease pathogenesis and therapeutic targeting.

A few studies have shown that systemic biofluids, such as plasma and serum, mirror local glaucomatous changes. These plasma and serum-based studies extend the understanding of glaucoma as a systemic disease. For instance, DIA-based plasma proteomics revealed differential responses to hypoxia, including enhanced immune activation and altered cholesterol metabolism. Apolipoproteins and inflammatory cytokines emerged as potential modulators of glaucoma risk, with anti-inflammatory signatures possibly offering protection in ocular hypertension (Langbøl et al., 2024). SOMAscan analysis identified neurofilament light chain and vascular-related proteins correlating with IOP, suggesting potential blood-based biomarkers for disease progression (Pessuti et al., 2025). Serum proteomics comparing primary open-angle glaucoma, pseudoexfoliation glaucoma, and controls identified a panel of 17 differentially expressed proteins validated across cohorts (González-Iglesias et al., 2014). These proteins, which are related to immune and inflammatory regulation, further support systemic contributions to glaucomatous pathogenesis and suggest subtype-specific differences. In addition, shotgun proteomics of peripheral blood mononuclear cells revealed widespread molecular dysregulation in glaucoma patients, including alterations in autophagy, mitochondrial metabolism, proteolytic balance, and immune signaling (Giammaria et al., 2025). These findings describe systemic immune involvement and mitochondrial dysfunction as key components of glaucoma, even outside the ocular compartment.

Pathway analysis using differentially expressed proteins reported in the above-discussed studies showed an overlap in complement/coagulation, neutrophil and platelet degranulation, oxidative stress, and ECM remodeling in almost all tissues (**Figure 3**). This indicates that glaucoma cannot be viewed solely as a localized retinal disorder; rather, these shared signatures reveal a systemic inflammatory and vascular dysregulation that converges with local retinal pathology. Complement activation and effector cell degranulation in plasma, peripheral blood mononuclear cells, and ocular fluids suggest persistent immune loops that compromise the BRB and prime retinal glia. Shared oxidative stress and ECM remodeling link systemic redox imbalance and biomechanical changes to local vulnerability. In contrast, the retina exhibits unique mitochondrial dysfunction, neurodegeneration, and phototransduction deficits, indicating the end-organ impact of systemic stress. Together, this supports a two-way model: systemic immune and vascular perturbations might amplify retinal injury, while retinal degeneration feeds back to activate circulating immunity. These findings describe glaucoma as a multifactorial disease shaped by reciprocal interactions between systemic and retinal pathways, with biofluids providing valuable windows into retinal pathology.

Crucially, correlating molecular signatures with clinical measures, such as IOP, visual field loss, and optical coherence tomography-derived structural changes, is essential for improving prognostic accuracy. While challenges remain, emerging studies increasingly link proteomic alterations with these clinical metrics. Longitudinal sampling of biofluids like tears and AH, combined with functional and imaging assessments, has begun to reveal dynamic proteomic shifts over the course of disease progression (Lee et al., 2022; Pessuti et al., 2025). Moreover, multi-omics integration, especially proteomic-metabolomic combinations, has helped clarify key glaucoma-related pathways involving lipid dysregulation, oxidative stress, and neuroimmune signaling (Rossi et al., 2019; Mok et al., 2024). Notably, multiplex profiling using Olink (Zhang et al., 2024a) technology identified aldehyde dehydrogenase 1 family member A1 (ALDH1A1) as a promising diagnostic marker, while multiple reaction monitoring-based analyses (Adav et al., 2018) confirmed complement activation in treated primary open-angle glaucoma patients.

Nonetheless, the precise alterations, heterogeneity, and intercellular interactions of retinal glial cells remain incompletely understood. Immune-magnetic isolation in experimental glaucoma models has highlighted astrocytic upregulation of immune responses, providing a foundation for glial-specific analyses in human tissues. Such approaches could elucidate layer- and cell-specific pathways, improving mechanistic understanding of glial contributions to neuroinflammation and glaucoma progression (Tezel et al., 2012). Advanced spatially resolved proteomic techniques, such as imaging-mass spectrometry (Bowrey et al., 2016), offer the potential to map protein expression within the intact retinal architecture, allowing researchers to link glial activation states with specific retinal layers, local microenvironments, and neighboring neuronal or vascular compartments. Such approaches could uncover layer-specific glial responses, identify microdomain-specific signaling pathways, and reveal how MG, astrocytes, and microglia coordinate during neuroinflammatory and degenerative processes. Ultimately, spatial proteomics could provide mechanistic insights that are unattainable with conventional bulk tissue analyses, bridging the gap between molecular profiling and functional retinal pathology. Further help in developing a deeper understanding of the role of glial cells in glaucoma progression.

**Figure 4:**
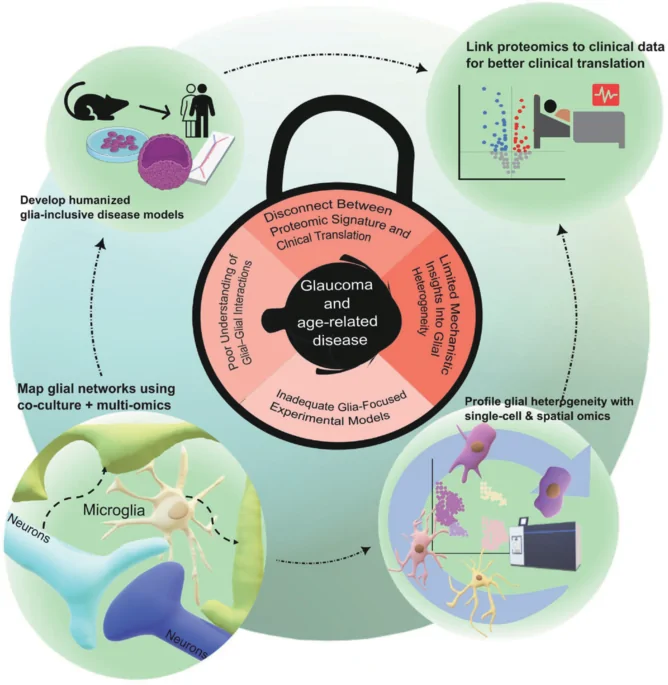
Integrating models and proteomics of retinal glial cells in retinal disease. Retinal glial cells actively drive disease progression, yet their diverse and disease-specific functions remain poorly understood. Integrating glial-focused models with high-throughput proteomics can uncover mechanistic insights, facilitate translational research, and enable the discovery of clinically relevant prognostic biomarkers. Vectors adapted from Servier Medical Art (https://smart.servier.com/), licensed under CCBY 4.0 (https://creativecommons.org/licenses/by/4.0/).

Looking ahead, future research must prioritize larger, longitudinal, and multi-center studies to validate these findings. Incorporating machine learning for biomarker selection, stratifying patients based on molecular endotypes, and aligning proteomic data with real-world clinical information will be crucial for implementing precision medicine approaches. Expanding beyond proteomics into multi-omics frameworks will enable a systems-level understanding of disease, while single-cell transcriptomics (Urrutia-Cabrera and Wong, 2020; You et al., 2021) and spatial profiling (Lalman et al., 2025) will provide unprecedented resolution of glial and immune heterogeneity within retinal niches. Additionally, the expanded use of minimally invasive sampling, such as plasma, could enable better diagnosis and more accessible disease monitoring. Translating these molecular insights into point-of-care diagnostics and personalized therapies hold transformative potential for glaucoma management and could help mitigate the global burden of irreversible vision loss (**Figure 4**).

## Conclusion

Retinal glial cells, including MG, astrocytes, and microglia, have emerged as key contributors to retinal neurodegeneration across major ocular diseases, including glaucoma, AMD, and DR. In the diseased state, however, these cells undergo profound functional and morphological reprogramming (Fletcher et al., 2008). Glial cells are not merely passive responders to neuronal injury, as has long been assumed. Rather, they are involved in the dynamic regulation of the retinal microenvironment, capable of both sustaining neuronal homeostasis and actively driving pathology, depending on the context and duration. Glial activation and immune responses are triggered by different triggers in glaucoma (elevated IOP, oxidative and metabolic stress, and mitochondrial dysfunction), AMD (chronic oxidative injury, complement dysregulation, and lipid accumulation), and DR (hyperglycemia-induced metabolic stress, vascular leakage, and ischemia). Across these diseases, shared intracellular pathways, including TLR, NF-κB, MAPK, JAK/STAT, and inflammasome signaling, regulate cytokine release, complement activation, and neuroimmune crosstalk. Meanwhile, interactions among glial subtypes modulate the magnitude and persistence of inflammatory responses, although these communication networks remain poorly defined. Importantly, it is the persistence, rather than the initiation, of these signalling cascades that determines whether glial responses remain adaptive or transition into maladaptive states, a challenge further compounded by the chronic nature of retinal diseases.

Despite growing evidence implicating glial cells in disease progression, strategies for vision restoration have historically focused on neuronal regeneration. However, neuronal health is intrinsically dependent on the surrounding microenvironment, which is largely shaped by the metabolic, inflammatory, and trophic support provided by glial cells. Key unresolved questions include whether disease-associated glial cells can revert to homeostatic states, the stages of disease at which such reversibility remains possible, and how effectively glial-modulating strategies can be translated to human patients.

Nonetheless, significant challenges remain. Experimental models provide mechanistic insight but often fail to capture the full complexity of human retinal disease. Rodent models, laser-induced CNV, and streptozotocin- or Akita-based mice models reproduce specific aspects of pathology but incompletely model chronic disease dynamics. In contrast, human-relevant systems, induced pluripotent stem cell–derived retinal organoids, ex vivo explants, and organ-on-chip platforms, better model glial–neuronal–vascular interactions, offering improved translational potential. Nevertheless, these systems remain underutilized for interrogating glial-specific mechanisms, and few studies have systematically examined glial functional states or transitions within such models.

Another limitation is the lack of well-defined molecular markers that reliably distinguish protective from pathogenic glial phenotypes across disease stages. Proteomic profiling using patient biofluids has revealed signatures of oxidative stress, mitochondrial dysfunction, complement activity, and inflammation. However, studies differentiating cell populations based on functional markers are limited. A critical unmet need in the field is the identification of robust molecular markers that can serve as reliable prognostic indicators. Achieving this will require larger patient cohorts with longitudinal follow-up to capture the temporal dynamics of glial phenotypes and their relationship to disease progression. Proteomic approaches provide a powerful framework to address this, enabling comprehensive profiling of cytokines, autoantibodies, and tissue-specific proteomes from retinal tissue, AH, vitreous humor, and peripheral blood plasma. Importantly, glaucoma and other retinal diseases exhibit systemic involvement, suggesting that systemic biomarkers may reveal novel prognostic indicators. Advancing research in this direction could enable predictive, personalized strategies, offering critical insights into both local retinal pathology and broader systemic contributions.

Overall, a strategic shift toward human-derived tissues and integrative, multi-omics frameworks represents a pivotal inflection point for the field. This strategy facilitates a deeper mechanistic understanding, the identification of prognostic biomarkers, and the development of novel therapeutic targets. Therapeutically, the focus should shift beyond symptomatic control toward modulating glial dysfunction, including limiting chronic microglial activation, restoring microglial metabolic and antioxidant capacity, and fine-tuning astrocytic reactivity. Emerging small molecules, biologics, and gene-editing approaches aim to harness these pathways, offering the potential for precision therapies that slow or halt retinal neurodegeneration across glaucoma, AMD, and DR.

In conclusion, the next frontier in retinal neurodegeneration lies in shifting the lens from neurons alone to the glial networks that sustain them. Glia-centered models, longitudinal human studies, multi-omics integration, and targeted modulation will together pave the way for precision medicine approaches capable of slowing or halting vision loss in glaucoma and other diseases.

## Additional files:

***Additional Table 1:*** *Summary of glial cell responses in health and disease.*

***Additional Table 2:*** *Summary of humanized models for assessing glial cells in retinal diseases.*

## Data Availability

*All relevant data are within the paper and its Additional files.*
